# Seeking treatment for symptomatic malaria in Papua New Guinea

**DOI:** 10.1186/1475-2875-9-268

**Published:** 2010-10-06

**Authors:** Carol P Davy, Elisa Sicuri, Maria Ome, Ellie Lawrence-Wood, Peter Siba, Gordon Warvi, Ivo Mueller, Lesong Conteh

**Affiliations:** 1 Papua New Guinea Institute of Medical Research, Goroka, Eastern Highland Province, Papua New Guinea; 2 School of Population Health, University of Adelaide, Adelaide, South Australia; 3 Barcelona Centre for International Health Research (CRESIB), Hospital Clínic/IDIBAPS, Universitat de Barcelona, Barcelona, Spain; 4 CIBER Epidemiologia y Salud Publica (CIBERESP), Barcelona, Spain; 5 School of Psychology, Flinders University, Adelaide, South Australia; 6 Swiss Centre for International Health, Swiss Tropical and Public Health Institute, Basel, Switzerland; 7 Institute for Global Health, Imperial College London, London, UK

## Abstract

**Background:**

Malaria places a significant burden on the limited resources of many low income countries. Knowing more about why and where people seek treatment will enable policy makers to better allocate the limited resources. This study aims to better understand what influences treatment-seeking behaviour for malaria in one such low-income country context, Papua New Guinea (PNG).

**Methods:**

Two culturally, linguistically and demographically different regions in PNG were selected as study sites. A cross sectional household survey was undertaken in both sites resulting in the collection of data on 928 individuals who reported suffering from malaria in the previous four weeks. A probit model was then used to identify the factors determining whether or not people sought treatment for presumptive malaria. Multinomial logit models also assisted in identifying the factors that determined where people sought treatments.

**Results:**

Results in this study build upon findings from other studies. For example, while distance in PNG has previously been seen as the primary factor in influencing whether any sort of treatment will be sought, in this study cultural influences and whether it was the first, second or even third treatment for a particular episode of malaria were also important. In addition, although formal health care facilities were the most popular treatment sources, it was also found that traditional healers were a common choice. In turn, the reasons why participants chose a particular type of treatment differed according to the whether they were seeking an initial or subsequent treatments.

**Conclusions:**

Simply bringing health services closer to where people live may not always result in a greater use of formal health care facilities. Policy makers in PNG need to consider within-country variation in treatment-seeking behaviour, the important role of traditional healers and also ensure that the community fully understands the potential implications of not seeking treatment for illnesses such as malaria at a formal health care facility.

## Background

Where, and if people seek treatment for symptoms of malaria is influenced by many factors, including individual perceptions, social norms, contextual constraints, institutional systems and economic circumstances [1]. By identifying treatment- seeking patterns and understanding the influences that are likely to shape a person's behaviour in each context, policy makers will be better able to make informed decisions about resource and infrastructure allocation [2].

For policy makers in PNG, identifying and understanding where people go to obtain treatment for symptoms of malaria is extremely important for two reasons. First, malaria is 'the leading cause of illness and death' [3]. Furthermore, treating this disease places a particularly heavy burden on the public health care system with 1.5 million outpatient visits in 2003 [4]. In addition, like many low-income countries, limited government revenue must be distributed between various competing interests including education and infrastructure, in addition to providing health care services [5,6]. Policy makers in PNG must, therefore, plan and allocate resources efficiently in order to reduce the impact of malaria and also to improve the health of the population as a whole [7].

There has been a significant amount of socio-behavioural research on treatment-seeking for malaria, which highlights factors influencing choice of provider [1,8-13]. However, the majority of these studies have focused on sub-Saharan Africa [12-17]. Nevertheless, various interconnecting consumer, illness and even provider characteristics have already been identified as influencing to some degree a person's treatment-seeking behaviour (TSB) [18]. From the health care consumers perspective, gender has been shown to not only affect the risk of getting malaria but also influence the type of treatment that a person will seek for themselves and in the case of mothers, for their family [19]. An individual's past experience may also influence TSB, with people who have already dealt with various malaria episode being less likely to seek treatment from a public health care facility [20]. Additional factors that have been identified as potential determinants of malaria treatment-seeking also include the size of the family, type of work undertaken [21,22], age [15], education and wealth [16].

The perceived characteristics of the illness have also been shown to affect where people go for treatment. In many cases, for example, traditional remedies are used in conjunction with biomedical treatments for malaria treatment [20] suggesting that illness may simultaneously be perceived in a variety of different ways. Therefore, choices of treatment are dependent not just on the strength of local traditions [23], but also on the person's knowledge of biomedical malaria treatments [24].

Lastly, the characteristics of the provider are also believed to be an important influence on an individual's TSB. Research into provider characteristics has tended to focus primarily on issues related to facility charges, demonstrating in some cases that people prefer cheaper options [25]. However, there are also indications that accessibility of appropriate facilities and medication [21], time taken and cost of travel [15], as well as the perceived effectiveness of treatment influence where and when people seek treatment for malaria [20].

In PNG, malaria treatment is generally available at any of the four types of public health care facilities. PNG has 19 provincial Hospitals, with the principle hospital in the capital, Port Moresby General Hospital. The health centre is the largest of the primary health care facilities and is often staffed by a health extension officers as well as several nurses and community health workers (CHWs). Larger health centres not only provide outpatient services but may also have inpatient facilities [4,26]. Sub-health centres are slightly smaller than health centres and are usually staffed by at least one nurse as well as several CHWs. Aid posts are the smallest of the public health care facilities and are often staffed by one CHW who is able to provide only basic first line treatment.

A fee for service is payable at many of these public health care facilities, but there is no government policy on how much should be charged. For example, some hospitals do not charge for inpatient paediatric services, while many health centres, sub-health centres and even aid posts impose a fee for service. The quality of services available to people seeking treatment varies significantly between facilities. For example, while health facilities in the New Ireland province of PNG were adequately stocked for an average of 81% of the year, facilities in West New Britain only had access to essential medications for approximately 38% of the year [27].

People are also known to seek treatment outside of the public health sector [15]. In PNG, privately owned pharmacies for example offer malaria diagnostic services and also sell a range of malaria treatments over the counter. In addition, for many people in PNG, traditional healers who may provide a variety of remedies in the form of herbs and/or spiritual healing are also an acceptable option for treating the symptoms of malaria [28]. In these circumstances, the costs associated with seeking treatment are generally worn by the patient and/or traditional healer, and do not directly impact on government spending.

One of the few malaria studies which has considered TSB in PNG, identified distance from a health facility, age and sex as factors that affected whether people in the Wosera region of the East Sepik sought treatment [29]. However, this study did not capture the use of providers outside of the public health care sector. Likewise, an earlier study, limited to treatment-seeking at aid posts in the Madang Province, found that perceptions pertaining to illness, distance from the facility and costs associated with getting to and accessing treatment all contributed to when and if treatment was obtained [30].

One study conducted in the PNG province of Bougainville did consider both formal and informal health care providers as well as the possibility that individuals may seek more than one type of treatment during an illness episode [31]. Proximity, costs and perceived effectiveness of treatment were discovered to be the most important factors in determining what type of treatment people sought [32,33]. However, the study only interviewed participants from one region of PNG and multiple disease groups were included.

In comparison, this article specifically focuses on malaria and considers the potential that individuals may engage with multiple public as well as private health providers during any one illness episode. In addition because of the geographic, cultural and linguistic variety that exists in PNG [34], it compares the TSB of two diverse regions in order to understand how factors which could impact on an individual's TSB, may themselves be influenced by cultural and contextual features.

Results from this treatment-seeking study should not only provide a broader understanding of the influences on treatment-seeking decisions for malaria in PNG, but also make available information, which could assist policy makers to improve service delivery. In particular, the treatment-seeking study aimed to both identify where individuals go to obtain treatment for malaria, if at all, as well as understand what factors influence their choices.

## Methods

This analysis draws on both qualitative (health-seeking behaviour) and quantitative data to better understand TSB in PNG. While these approaches may have different methodological and theoretical underpinnings, it is not uncommon for these types of data to be combined within a single study [35].

### Study sites and population

Two study areas were chosen because they were geographically, culturally and linguistically different. In addition, the malaria ecology between the two sites also varies. While both study sites are, considered to be primarily rural in nature, the size and distance to the nearest urban facility differs between the two.

**Figure 1:**
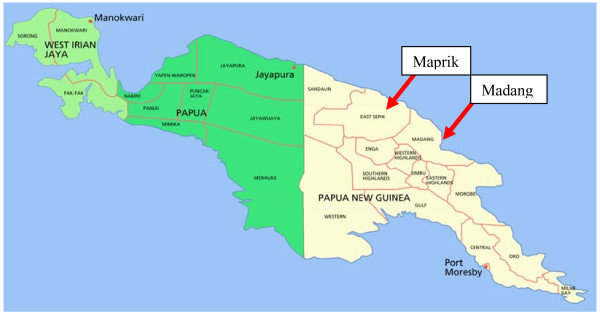
**Map Identifying Data Collection Sites (*Map of the Island of New Guinea *2006)**. This image has been copied under a GNU Free Documentation License, Version 1.3; with no Invariant Sections, no Front-Cover Texts, and no Back-Cover Texts.

The first of the two sites is situated within 20 kms from the provincial capital of the Madang Province. The provincial capital, also named Madang, is a coastal town situated on the north coast of PNG (refer Figure 1). The township Madang has a population of approximately 35,000. People living within this site would generally have more access to public and private services. Malaria in this area is hyperendemic with limited seasonality [36]. Both *Plasmodium falciparum *and *Plasmodium vivax *are common and the burden of disease is concentrated in young age groups [36,37].

The second study site is situated near the local administrative centre in Maprik which is approximately 70 kms inland (West South West) from the nearest provincial capital of the East Sepik Province, Wewak (refer Figure 1). The township of Maprik has a population of approximately 5,000. As Maprik does not have the same characterisitics as Madang, people living within this study site would not have the same access to public and private services. In addition, malaria in this study site has traditionally been holoendemic with all year round high transmission of both *P. falciparum *and *P. vivax *[38,39]. However, in recent years a significant reduction in the prevalence of *P. falciparum *in rural areas has been observed [40].

The study population consisted of individuals who met particular study criteria. To be considered, individuals had to have been residing in either of the selected sites for a minimum of at least eight weeks prior to the survey and have been diagnosed with malaria and/or experienced fever in the previous four weeks. Fever was included within the study criteria because in PNG laboratory tests and rapid diagnostic tests are often not available and therefore fever is considered to be a strong indication of malaria [8]. This study, therefore, includes confirmed and presumptive malaria episodes.

### Study design and sample

Research has previously shown that distance [29] was a significant factor in determining TSB of Papua New Guineans. Yet distance form the nearest health centre also depended on the geographical terrain. In some cases people in villages closest to a health centre found accessing the facility more difficult in comparison to people in villages further away. This cross sectional study, therefore, identified three groups of villages, within each study area, based on the average time it would take for a healthy person to walk to their nearest health centre.

• Group One (G1) - villages less than half an hour walk

• Group Two (G2) - villages between 1 - 2 hours walk

• Group Three (G3) - villages greater than 3 hours walk.

In January 2008, 14 individuals who had recently graduated from year 12 were selected from local villages near each of the study sites to collect data for the study. Employing local data collectors was particular beneficial for three reasons. First, because many of the villages were spread across a wide area, geographical knowledge was important. Second, being able to speak the local language was often not only appropriate, but appreciated by study participants. In addition, the local data collectors were particularly motivated to carry out the data collection phase, believing that it would provide information which in the long run could benefit their communities. These data collectors received a one-week training on the principles of informed consent, and conducting treatment-seeking surveys.

The survey tool used by the data collectors captured data pertaining to initial and any subsequent treatment/s for each episode of malaria, whether or not this treatment was obtained from a formal health care facility. Part one of the survey incorporated demographic questions on the age, sex and family status for each individual who met the study criteria. Part two collected data pertaining to choice of provider(s), the preferences associated with these choices as well as distance to the chosen provider. While categories, such as hospital, health or sub-health centre and traditional healer, were provided for ease of recording, particular emphasis during training was placed on the importance of not prompting the informant in any way. If an answer did not fit into the broad categories provided on the survey tool, data collectors wrote out the answer in full.

The sample size was calculated using the following formula: *n = Z^2^pq/e^2 ^*where confidence level (Z) = 1.96, variability (p) = 0.50, (q) = 0.50, precision (e) = 6%. That is, n = (1.96)^2^(0.5)(0.5)/(0.06)^2 ^= 267, being the minimum number of "houses" that needed to participate in order to have a representative sample according to parameters chosen. In particular, because of the presumed high variability of population across sites and villages chosen, *p *and *q *were set at their maximum possible level, of 50% [41].

Houses where at least one member had experienced symptoms of malaria including fever and/or convulsions in the previous four weeks were included in the study. Because of work and social commitments it was not possible to personally interview everyone identified as having had a suspected episode of malaria. Therefore, one adult member from each household was asked to provide information on behalf of the other household members. Data on the TSB of 433 individuals from 259 households in Madang, and 495 individuals from 289 households in Maprik, were obtained.

Field workers were trained to select houses from all geographical parts of the village, as the position within the social structure was to some extent reflected by the geographical position of the house within the village. That is, people living in houses on the outskirts of the village were believed to be lower within the village social structure, in comparison to those living within the centre.

### Ethical considerations

Prior to commencing, a full study protocol and survey tool was submitted to and approved by both the PNG Institute of Medical Research (IMR) Internal Review Board and the PNG Medical Advisory Committee. To ensure the highest possible ethical and operational research standards the study was also conducted in accordance with the GCP E6 Guidelines [42].

### Model and analysis

Similar to other treatment-seeking studies conducted in low-income countries [16,43-45], two multivariate models aimed at analysing the determinants of malaria TSB were estimated, each testing a different hypothesis. Behind both models lay the neoclassical utility theory assumption that people will generally choose to maximize their utility function [46]. First, a probit model was used to analyse the factors determining whether or not people sought treatment for presumptive malaria. Probit can be seen as a latent variable model (Formula 1).

$$\begin{array}{l} Y^{*}=x^{'}\beta +\varepsilon \\ Y^{*}\text{ represents the latent variable}\\ Y=\left\{\begin{array}{l} 1ifY^{*}>0\\ 0ifY^{*}\leq 0 \end{array}\right\} \end{array}$$

#### Formula 1 - Probit Formula

The sample size used in the probit model consisted of all 928 observations collected (433 individuals from 259 households in Madang, and 495 individuals from 289 households in Maprik). If the utility people received from seeking treatment in case of fever (Y*) was positive, then they did seek treatment (*Y = 1*); if the utility people received from not seeking is 0 or negative, then they did not seek treatment (*Y = 0*). *X*' is a vector of explanatory variables determining the utility people take from the choices they made.

The second analysis was conducted using multinomial logit models to identify the factors that determined where people sought a first, and if relevant, a second treatment for each reported episode of presumptive malaria. A third treatment is included in data collected but because of the small sample (51 observations) this was not included in the multinomial logit analysis. Data collected on the third treatment will be shown only from a descriptive point of view.

Multinomial logit is a non linear model, estimated by maximum likelihood techniques that can be expressed in the following way (Formula 2):

$$P(Y_{i}=j)=\frac{\exp(X_{i}\beta_{j})}{1+\sum_{j}^{J}\exp(X_{i}\beta_{j})}$$

#### Formula 2 - Multinominal Logit Formula

Each individual (*i*) faces a set (*j*) of options. The probability they choose the *j*^th ^option is given by the Multinominal Logit Formula (Formula 2). The probability that option 0 (reference option) is chosen is given by Formula 3.

$$P(Y_{i}=0)=\frac{1}{1+\sum_{j}^{J}\exp(X_{i}\beta_{j})}$$

#### Formula 3 - Multinomial logit formula with *j = 0*

*X *is a set of independent variables potentially explaining the choice. People choose one of the options *j *rather than the option 0 if their utility (explained by *X*) in choosing *j *is higher than the utility of choosing *0*. For instance, People would choose to go to the aid post (one of the option *j*) rather than going to the health centre (*0*) if they can get higher utility.

On the basis of initial analyses, a reference option of choice, corresponding to *j = 0 *in Formula 3 was selected prior to analysing data through the multinomial logit model. "health centre" was chosen as the reference for interpreting where people sought treatment; that is a person's choice to seek treatment was always explained in comparison to the potential choice of going to a health centre. The "health centre" was chosen as a reference point for two reasons. First, because it is part of the formal health care systems it is considered to be a stable and measurable comparison. Second, in this context, health centres represent the treatment source of choice. This is primarily because health centres are more likely to offer a higher quality of care with more sophisticated diagnostic services and a greater range of drugs than a sub-health centre or aid post, while still being more accessible than hospitals which are generally only situated in major provincial capitals. Therefore, it could be presumed that patients would choose to go to a health centre if at all possible and the question becomes "why do people choose any type of treatment instead of going to the health centre?"

Data from questionnaires regarding reasons for treatment choice were grouped into 10 categories (see Table 1), and a series of univariate tests (Pearson χ^2^) were conducted to determine which reasons predicted treatment choice. Only those variables that were significant were then included as predictors in the multinomial logit model.

**Table 1:** Definition of categories

Category	Definition
*1*. Site	Living in a village which is situated within either the Madang or Maprik region
*2*. Group	G1 - villages less than half an hour walk G2 - villages between 1 - 2 hours walk G3 - villages greater than 3 hours walk.
*3*. Age	Reported age of individual seeking treatment (as a continuous variable)
*4*. Access	Participant reports which relate to ability to access a treatment provider
*5*. Perception	Participant reports which relate to quality of the treatment, both positive and negative
*6*. Availability of Medicine	Participant reports which relate to availability of medicine
*7*. Finding a Cure	Participant reports which relate to a need to seek treatment in order to find a cure
*8*. Focusing on the Illness	Participant reports which focus on the illness rather than identifying a way of treating it
*9*. Minutes	Minutes taken to travel to a treatment as reported by the participant
*10*. Other	A small number of participant reports which primarily related to seeking a particular treatment "because they usually do" or "because they were referred there" were grouped under this category

## Results

The following results relate to the number of reported observations of presumptive malaria. For these reported observations, the mean age for males who had experienced symptoms of malaria was sixteen (SD = 18.35) and the mean age for females was fifteen years (SD = 18.63). Of the total 928 recorded incidences of symptomatic malaria 696 were reported to involve children, 196 involved parents, 22 involved grandparents and 14 involved other relatives or friends residing in the household. Of the total number of observations collected, thirteen were discarded due to a missing control variable, leaving a total sample size of 915. Nine percent of this sample (n = 81) chose not to seek any treatment for their malaria symptoms, while 91 percent (n = 834) reported seeking at least one type of treatment.

### Factors influencing whether or not to seek treatment

The only significant predictor of TSB was the distance category of the village participants lived in (G1, G2, or G3) (see Table 2). Specifically, participants living in G3 villages (those furthest away from a health facility) were less likely to seek treatment, with living in one of these villages reducing the probability of seeking treatment by 7%. The fact that this was not the case for G2 villages highlights the non-linearity of the effect of distance on the choice to seek treatment.

**Table 2:** Results from probit regression

Control variables	Coefficients	Marginal Effects
**Village groups**		
Ref: G1		
G2	-0.07 (-0.44; 0.30)	-0.01 (-0.06; 0.04)
G3	**-0.47** (-0.80; -0.14)**	**-0.07** (-0.11; -0.02)**
**Age**	0.00 (-0.01; 0.01)	0.00 (-0.00; 0.00)
**Gender (1 = Male)**	-0.01 (-0.33; 0.31)	-0.00 (-0.05; 0.04)
Age*Gender	-0.00 (-0.01; 0.01)	-0.00 (-0.00; 0.00)
Constant	**1.76** (1.41; 2.12)**	
Observations	915	915

Robust Confidence Interval (95%) in brackets; *significant at 5%,**significant at 1%

Although data pertaining to why participants did not seek treatment was not included in the probit regression due to the small sub-sample of total observations they represent (n = 71), it is briefly discussed here. For participants in G1 villages (n = 13) after lack of motivation (50%), distance (14%) along with illness severity (14%), was the equal second most important reason for not accessing treatment.

For participants in G3 villages (n = 44), the most common reasons for not seeking treatment were illness severity (27%) (their illness was not severe enough), lack of motivation (25%), treatment cost (20%) (they did not have enough money), followed by distance (16%) (treatment too far away).

Participants in G2 villages (n = 14) sited 'other' reasons (50%) including 'the fever had only just started', didn't like going to the health centre or taking medicine or believing it will stop without help. Other common reasons for not seeking treatment included their illness not being severe enough (29%) were the most common explanations provided for not seeking treatment, followed by lack of motivation (14%) and not trusting the treatment (7%).

### Where do participants seek treatment?

Descriptive analyses indicated that the most common treatment choices for first, second and third treatments were health and sub-health centres. This finding was consistent across both sites. Any type of home supply (including previously acquired or left-over medications) were less likely to be used, and when they were used, this occurred more often in the case of third treatments. Hospitals and pharmacies were also less popular treatment sources for first, second and third treatments, while shops, street traders and neighbours were rarely used.

### Treatment Progression

Of observations where at least one episode of treatment was sought (n = 834), approximately 28% (n = 234) went on to seek a second treatment for the same episode of symptomatic malaria. While people seeking a second treatment were still more likely to use a health centre than other treatment provider, there was a significant difference in types of treatment sought between Treatment 1 and Treatment 2, *X² *(1, *n *= 42) = 121.89, p < .001. Results from the multinominal logit model also showed that the reasons why participants choose a particular type of treatment changed as their illness progressed (Table 3).

**Table 3:** Significant Reasons as to why a treatment option was chosen

Reasons for treatment seeking										
	Ref: Health Centre	Site	Age	Accessibility of Provider	Perception of Provider	Drug Availability	Wanting to Get better	Being sick	Minutes	Other
First treatment (n = 834)	Hospital	**-1.89** [-3.05; -0.72]**	0.01 [-0.00; 0.03]	**-2.12** [-3.51; -0.74]**	**-2.23** [-4.29; -0.16]*	**-1.81** [-3.59; -0.02]*	**-2.78** [-3.59; -0.02]*	-0.18 [-2.15; 1.79]	0.00 [-0.01; 0.01]	**#**
	Sub Health Centre	**1.09** [0.47; 1.72]**	0 [-0.01; 0.01]	-0.40 [-1.09; 0.28]	**-0.98** [-1.66; -0.30]**	-0.22 [-0.87; 0.43]	-0.01 [-0.83; 0.81]	-0.37 [-1.21; 0.46]	**-0.02** [-0.02; -0.01]**	**#**
	Aid Post	-1.93 [-3.93; 0.07]	**0.03** [0.01; 0.05]*	-1.42 [-3.13; 0.29]	0.66 [-0.26; 1.58]	-0.84 [-2.35; 0.67]	0.09 [-1.16; 1.34]	0.17 [-1.65; 1.98]	-0.01 [-0.03; 0.00]	**#**
	Pharmacy/Shop/ Kiosk/Street Trader	**-2.47** [-4.76; -0.18]*	0.02 [-0.01; 0.05]	**-1.70** [-2.93; -0.46]**	0.01 [-1.20; 1.22]	**-2.36** [-3.71; -1.02]**	-1.40 [-3.87; 1.06]	**-38.38** [-39.74; -37.03]**	**-0.10** [-0.18; -0.02]*	**#**
	Neighbour	-0.56 [-1.60; 0.48]	0.01 [-0.02; 0.03]	**-2.24** [-3.32; -1.17]**	0.60 [-0.44; 1.64]	**-3.34** [-4.85; -1.84]**	-0.48 [-1.76; 0.80]	-0.63 [-2.32; 1.05]	**-0.19** [-0.24; -0.13]**	**#**
	Traditional Healer	**-2.62** [-4.76; -0.48]*	-0.01 [-0.04; 0.02]	**-1.75** [-3.08; -0.42]**	**-37.00** [-38.52; -35.47]**	0.44 [-1.08; 1.95]	**-37.39** [-39.50; -35.28]**	0.4 [-3.36; 4.16]	**-0.98** [-1.50; -0.45]**	**#**
Second treatment (n = 231)	Hospital	-0.06 [-1.16; 1.04]	**#**	0.68 [-0.53; 1.89]	**#**	**#**	**#**	-0.39 [-1.69; 0.92]	**3.47** [1.56; 5.38]**	**0.01** [0.00; 0.02]*
	Sub Health Centre	1.00 [-0.06; 2.06]	**#**	-1.29 [-2.88; 0.31]	**#**	**#**	**#**	0.15 [0.84; 1.14]	**-34.42** [-36.08; -32.76]**	0.00 [-0.01; 0.01]
	Aid Post	**-34.67** [-35.82; -33.51]**	**#**	0.38 [-1.61; 2.36]	**#**	**#**	**#**	0.74 [-0.58; 2.07]	**-34.14** [-35.80; -32.49]**	0.01 [-0.01; 0.02]
	Neighbour	0.36 [-0.30; 1.03]	**#**	0.33 [-0.42; 1.08]	**#**	**#**	**#**	**-2.32** [-3.48; -1.16]**	1.55 [-0.18; 3.27]	0 [-0.01; 0.01]
	Traditional Healer	-0.40 [-1.97; 1.18]	**#**	**2.47** [0.76; 4.18]**	**#**	**#**	**#**	**-34.94** [-35.94; -33.94]**	2.33 [-0.08; 4.73]	0.01 [-0.00; 0.02]

Estimated multinomial logistic regression coefficients are reported and robust 95% CI are in brackets; (regression was clustered to adjust standard errors for intra-group correlation. Cluster was the "house" as sample unit in the survey conducted)*significant at 5%; **significant at 1%.

#Not included because no statistically significant relation with the dependent variable in the univariate analysis, p >.10

#### First treatment

The choice of provider varied between the two sites (see Table 3). Participants living in Madang, compared to those in Maprik were more likely to visit a health centre for malaria treatment as opposed to a hospital, shop or traditional healer. Likewise, the people living in Madang, compared to those in Maprik, were also less likely to visit a sub-health centre than a health centre. However, negative perceptions about the provider were also a significant reason for selecting another type of service.

Participants gave a number of different reasons for choosing a particular treatment provider. Those participants who focused on the illness rather than seeking a cure, for example, were less likely to visit a pharmacy, shop or kiosk compared to a health centre, although this did not appear to influence their choice of other providers. The reported time taken in minutes to travel to a treatment provider was another predictor for choosing a health centre over a sub-health centre, a traditional healer or medicine from a shop or neighbour.

Accessibility of treatment providers, seeking a cure, and a focus on the illness were also predictors associated with choosing a health centre over all other treatment providers except aid posts. For example, the services provided by a hospital in comparison to a health centre, were perceived to be harder to access, associated with less favourable treatment quality and were less likely to improve health status.

#### Subsequent treatment

The reasons why participants choose to go to a particular treatment provider differed for second treatments. While living in Maprik was significant for explaining the overall choice of health or sub-health centre as a first treatment option, living in Maprik was only a significant factor for choosing between a health centre and an aid post, if a second treatment was required.

In comparison to quality of service issues, including the availability of drugs and accessibility of providers seen in the first treatment, participants were more likely to choose the health centre as their second treatment provider for 'other reasons'; which primarily revolved around being "where they usually went" or because "they were told to go there".

There was also some association between choosing a health centre for subsequent treatments and the objective time in minutes it took to travel there. Surprisingly, however, accessibility was not reported as a significant subjective reason for choosing the health centre as a second treatment option. In contrast, participants did report choosing traditional healers over a health centre for a second treatment because they believed them to be more accessible.

## Discussion

One of the more important criteria for planning health services is knowing where, or even if, people seek treatment [47]. However, little attention has been paid to understanding malaria TSB in PNG. This article not only provides a better understanding of what treatments are sought, be they based on biomedical and/or traditional belief systems, but also what factors contributed to these treatment-seeking choices including how distance may not be a primary factor influencing TSB in PNG and how attitudes towards TSB may change as a malaria episode progresses.

### Factors influencing whether or not to seek treatment

At first glance the above results (Table 3) fully support the findings of previous treatment-seeking studies in PNG, which identified distance as a significant determinant of whether or not people seek treatment [29-31]. Participants in G3 villages being those further away from a health or sub-health centre were less likely to seek any sort of treatment, in comparison to those in G1 villages.

However closer analysis of the data suggested three reasons why distance per se, may not be the only factor contributing to these results. First, the effect of distance on the choice of seeking treatment in PNG, found in previous studies [29-31] is not linear. For example, although living further away from a health facility (G3 villages) appeared to be important, living closer to a health facility (G2 villages) did not mean that participants were more likely to seek any sort of treatment.

Second, participants living in G3 villages who chose not to seek treatment, not only decided against providers that were some distance away but also those who were relatively close at hand. If distance was the only factor involved in their decision, individuals would not have abandoned treatment altogether but instead sought help from an aid post or alternatively even a traditional healer.

Third, when participants were asked why they chose not to seek treatment they focused primarily on issues relating to lack of time, motivation and/or the belief that the illness did not warrant any particular action. Therefore, how important the individual perceived their illness to be in comparison to perhaps other factors including their social and family responsibilities appeared to be a bigger issue than distance.

There may also be a relationship between distance and perceptions of illness. The results from participants in G3 villages could indicate different perceptions and belief about illness in comparison to participants in G1 and G2. Because health centres are generally located in or near townships, being further away from a health centre may also indicate a degree of isolation. Isolation in turn encourages the development of distinct belief systems and patterns of behaviour [34], which are often reinforced within the isolated group through interactions with like-minded members [48,49]. In turn, these beliefs may influence how individuals react to an illness and even whether treatment is or is not sought.

### Where do participants seek treatment?

Overall, health centres in Madang and to a certain extent sub-health centres in Maprik were the treatment provider of choice when treatment for malaria was sought. Yet, unlike an earlier study in PNG [29], results from this research suggest that individuals did not necessarily choose these facilities in the first instance only because they were accessible. Instead, facilities were also chosen because they were perceived to be of better quality, the individual recognized they were ill or were seeking a cure. This suggests that along with accessibility, perceived quality and effectiveness of the service were also important.

It is interesting to note the preference for the health centres over the hospital. In many other settings the activity of 'bypassing' is reported, where people do not adhere to a referral system and travel longer distances to access care from higher level facilities or more costly providers in the first instance [50,51]. This does not seem to be the case in this study, as the hospital was not perceived to be the preferred source of treatment.

Much is also written in the literature about the large proportion of people accessing care outside government health facilities, with private providers seen as a reliable [52] and easily accessible supply of drugs [53-55]. In this study there was a clear preference for public health care facilities, notably health centres, over private providers such as pharmacies or shops selling anti malaria medication. One reason may be that anti malaria medication provided through public health care facilities in PNG is generally less expensive than that provided by shops or pharmacies. However, the findings from this study did not suggest that expense was a significant factor for choosing a formal health care facility. Instead, findings did suggest that people were less confident of receiving the drugs they needed at shops, compared to health centres. This perception that private retailers such as pharmacies, shops and kiosks were less likely to stock drugs is contrary to what is often reported in the literature [52,56].

### Treatment progression

Subsequent treatments for the same episode of malaria did not necessarily follow the patterns established in the first treatment. First, there were some changes to the preferred provider. While health centres and to some extent sub-health centres dominated in treatment one, hospitals and traditional healers were also providers of choice in seeking subsequent treatment.

The reasons why these choices were made also varied. Although living in Maprik was significant for explaining the overall choice of health or sub-health centre as a first treatment option, living in Maprik was only an important factor for choosing between a health centre and an aid post if a second treatment was required. Likewise, participants seeking subsequent treatments were more likely to choose a hospital over a health centre for 'other' reasons. This included using the hospital in the past or being told to go there by, for example, another health professional. Traditional healers were also more likely to be selected if the individual perceived them to be more accessible than other types of treatment providers.

While the probability of seeking services from a traditional healer rather than a health centre increases with access, a focus on the illness reduces the probability of seeking help from a traditional healer or neighbour. This suggests two things. First, traditional healers are only used if they are easily accessible. Second, people appear to distinguish between what can be treated by traditional medicine and being 'ill' which requires treatment in a formal health care facility.

### Beliefs and perceptions

Beliefs and perceptions pertaining to illness are important for determining where or even if a person will seek treatment [20,57,58]. For example, in Ghana pallor a symptom often associated with malaria is believed to result from a loss of blood and therefore serious enough to seek prompt treatment. In comparison, a study conducted in Uganda found that pallor was not considered to be serious and did not necessarily hasten treatment-seeking [59].

Three distinct groups of belief systems pertaining to illness have been identified within PNG [58]. First, sickness caused by sorcery or witchcraft. Second, sickness pertaining to the environmental or seasonal changes such as malaria or flu, and finally illness introduced by the 'white man', which primarily refers to diseases such as cancer and diabetes. If the first treatment at a formal health care facility fails, people may believe that it is not something that 'white man' can cure. Therefore, it is perhaps unsurprising then that people may in some cases choose to utilize traditional medicine. Yet as this study also demonstrates, if people believe they are truly ill they may also make more of an effort in subsequent treatment-seeking to seek help from a formal health facility.

For policy makers in PNG, this presents a particular difficult problem. Simply bringing health services closer to where people live may not necessarily always result in a greater use of formal health care facilities. Instead, a multi pronged approached may be required which recognizes the multi belief systems that do exist.

Policy makers in PNG have already moved towards a more flexible approach by introduction of the PNG National Policy on Traditional Medicine [60] which recognizes the contribution of traditional medicine to a community's health and well-being. However, more needs to be done. As a first step, formal health care facilities may need to seek ways in which they can work with rather than in opposition to traditional healers.

Community education programs could compliment this approach by encouraging people to also consider seeking treatment from formal health care facilities. One way of ensuring that these types of programs are effective is to involve community members in the development and implementation phases [61]. These local experts may be able to assist in ensuring that the information provided was contextually relevant, as well as act as advocates for the continued use of formal health care providers.

### Recommendations for further research

A primary strength of this study was the ability to collect and analyse data pertaining to entire malaria episodes rather than single treatments. Future studies may build upon this strength by considering the following limitations. First, incorporating data on previously identified influences on TSB, including direct financial factors such as treatment costs, user fees and household income [62-67], indirect costs of seeking treatment such as loss of income and time [1,62,67], urban or rural dwelling [15,63,67]; household size and the effects of overcrowding, [1,63,65,66]; caregiver characteristics such as literacy [63,15], previous treatment-seeking experiences, educational status and the severity of illness [67] will substantially add to the findings from this study.

Second, participants may have had some difficulty in providing accurate accounts of the experiences of other members of the household, particular within the four (4) week recall period used in this study. Lastly, while the analysis highlights the importance of within country variation, the extent that results from this study can be generalized to other areas of PNG needs further investigation.

## Conclusions

Papua New Guineans seek treatment for symptoms of malaria from a range of providers including those that exist outside of the formal health care system. Despite this complex and diverse context, understanding TSB in PNG may be crucial to the effective allocation of scarce health resources.

Findings from this study contribute to this understanding in three ways. First, it identified that differences in why and to some extent where people seek treatment may be influenced by whether or not they have previously sought treatment for the same malaria episode. Second, although distance is still a contributing factor, bringing health facilities closer to the end user may not necessarily result in higher use. Finally, the study demonstrates that patterns of treatment-seeking vary according to the particular context. Therefore, continual research is required to keep abreast of the demands placed on a country's formal health system.

## Competing interests

The authors declare that they have no competing interests.

## Authors' contributions

The study was conceived and designed by CPD, ES, IM and LC. CPD, and MO made a substantial contribution to the collection of data. The data was analysed and interpreted by CPD, ES, ELW, IM, GW and LC. CPD, ES and ELW drafted the paper, with substantial intellectual input into the final version from LC, ES and IM. All authors have approved the final manuscript.
